# Rate Distortion Theory for Descriptive Statistics

**DOI:** 10.3390/e25030456

**Published:** 2023-03-05

**Authors:** Peter Harremoës

**Affiliations:** GSK Department, Niels Brock, Copenhagen Business College, Nørre Voldgade 34, 1358 Copenhagen K, Denmark; harremoes@ieee.org

**Keywords:** rate distortion theory, quantizer, descriptive statistics, clustering, Gaussian mixture models, outlier detection, linear regression, Anscombe quartet, qibla, early Islam, 94A34, 62B10

## Abstract

Rate distortion theory was developed for optimizing lossy compression of data, but it also has applications in statistics. In this paper, we illustrate how rate distortion theory can be used to analyze various datasets. The analysis involves testing, identification of outliers, choice of compression rate, calculation of optimal reconstruction points, and assigning “descriptive confidence regions” to the reconstruction points. We study four models or datasets of increasing complexity: clustering, Gaussian models, linear regression, and a dataset describing orientations of early Islamic mosques. These examples illustrate how rate distortion analysis may serve as a common framework for handling different statistical problems.

## 1. Introduction

When Shannon initiated information theory in 1948, it became clear that lossless compression requires a kind of statistical analysis of the information source, in which the strings that are common, are assigned shorter code words than less common strings. In this way, any pattern in the information source can be used to compress data. Soon after, it was noted that there is an intimate relation between information divergence (KL-divergence) and the notion of sufficiency in statistics [1,2]. Since then, there have been a lot of interactions between lossless source coding and statistics [3].

It has been shown that under quite general circumstances, information divergence is more Bahadur-efficient for testing Goodness-of-Fit than, for instance, the classical $\chi^{2}$-statistic [4,5,6]. If data are discrete while the null-hypothesis may claim that the distribution is continuous, one can place data into bins and compare with the probability of these bins under the null-hypothesis. It should be noted that there are different notions of statistical efficiency and there are many special cases to be considered, and in some papers, the authors have concluded that although a straightforward application of entropy-based tests have good performance, there are cases where other tests are more efficient [7,8,9,10].

In the 1980s, J. Rissanen introduced the minimum description length principle as a new kind of statistical inference based on information theory [11,12,13]. With this kind of inference, a statistical model is not understood as a mechanism that has generated data but as a method for describing data. Information theory may be viewed as a theory of sequences that takes a position between Bayesian statistics that focuses on finite sequences and frequential statistics that focuses on infinite sequences. In information theory, the focus is on extendable sequences, i.e., finite sequences that are potentially prefixes of longer sequences [14]. This idea is quantified by Kraft’s Inequality.

Ideas from lossless source coding have also interacted with probability theory. For instance, most of the most important laws in probability theory can be reformulated like “entropy increases to its maximum” or “information decreases to its minimum”. This line of research was initiated by Linnik [15] with an information theoretic version of the central limit theorem, which was later improved [16,17,18]. Information theory has also interacted with ergodic theory [19,20], martingale theory [21,22], convergence of Markov chains [23,24], and the law of small numbers [25,26,27].

With the strong interaction between lossless source coding, statistics, and probability theory in mind, it is surprising that lossy source coding has had little effect on statistics. In this paper, we demonstrate that ideas from lossy source coding have the potential of unifying a number of classical methods in statistics, and, due to their flexibility, they can also be used to handle new problems in a non-trivial way.

### 1.1. Preliminaries on Rate Distortion Theory

Rate distortion theory was introduced by Shannon as a tool for lossy compression [28]. The goal is to compress an information source to a rate so that it could be sent through a communication channel with limited capacity. During the compression, some details in the dataset may be lost so that it is not possible to recover the information source exactly from the compressed source. The loss in quality due to compression is measured by distortion. Rate distortion theory was further developed and described in the textbook by Berger [29], and the elementary textbook by Cover and Thomas [30]. Rate distortion theory has had a significant impact on, for instance, image compression.

First, consider a random variable *X* with values in a *source alphabet* X and with distribution *P*. In principle, X could be any measurable set, but for our applications, X will typically be a dataset with *P* being the empirical distribution. During the compression, the source alphabet is mapped into a *reconstruction alphabet* by a *quantizer* $q:X\to \hat{X}$, and we obtain a random variable $\hat{X}=q\left(X\right)$. The reconstruction alphabet could be identical to the source alphabet, or it could be a subset of the source alphabet, or it could be a completely different set.

Next, we introduce a *distortion function* $d:X\times \hat{X}\to R_{0,+}$. If the reconstruction alphabet is a subset of the source alphabet, then the distortion function may be a metric or a function of a metric, but, in general, there are no restrictions on the functions that could serve as distortion functions.

**Example 1.**  *A Gaussian source has R as both source alphabet and reconstruction alphabet. As distortion function, one uses the squared Euclidean distance.*

For a quantizer *q* with $\hat{X}=q\left(X\right)$, the *rate* is defined as the entropy $H\left(\hat{X}\right),$ which can be written in terms of mutual information as

(1) $$H\left(\hat{X}\right)=H\left(\hat{X}\right)-H\left(\hat{X}\;\;|\;\;X\right)=I\left(X,\hat{X}\right).$$

The (mean) distortion is defined as $E\left[d\left(X,\hat{X}\right)\right]$. For each quantizer, one gets a distortion rate pair $\left(E\left[d\left(X,\hat{X}\right]\right),I\left(X,\hat{X}\right)\right)$ that can be plotted as a point in a coordinate system. A point that can be obtained as a distortion rate pair is said to be *achievable*.

Instead of looking at quantizers that act on single source letters, we may consider *vector quantizers*, which are quantizers acting on sequences. The rate of a quantizer $q:X^{n}\to {\hat{X}}^{n}$ is defined as $\frac{1}{n}H\left(q\left(X^{n}\right)\right)$. Let $x_{1}^{n}=\left(x_{1},x_{2},\ldots ,x_{n}\right)$ be a sequence of source letters and let ${\hat{x}}_{1}^{n}=\left({\hat{x}}_{1},{\hat{x}}_{2},\ldots ,{\hat{x}}_{n}\right)$ be a sequence of reconstruction letters. Then, the average distortion between the sequences is defined by

(2) $$d\left(x_{1}^{n},{\hat{x}}_{1}^{n}\right)=\frac{1}{n}\sum_{i=1}^{n}d\left(x_{i},{\hat{x}}_{i}\right).$$

Vector quantization will lead to new achievable distortion rate pairs. The set of points that are achievable by vector quantization is called the *distortion rate region*.

The lower convex envelope of the distortion rate region is called the *rate distortion function*. In general, one cannot get a low rate and low distortion at the same time and the distortion rate function gives the optimal trade-off between rate and distortion. Points on the graphs of the rate distortion function can be parameterized by either the rate or the distortion or the slope *s* of the function. For calculations, it is often simpler to parameterize the curve by its slope, which gives the unconstrained optimization problem of minimizing

(3) $$\frac{1}{n}I\left(X_{1}^{n},{\hat{X}}_{1}^{n}\right)-sE\left[d\left(X_{1}^{n},{\hat{X}}_{1}^{n}\right)\right].$$

To find the optimal quantizer *q* for a specified slope is NP-hard, but for applications, we just need a good approximation. We may look at a related problem where the quantizer $q:X\to \hat{X}$ is replaced by a Markov kernel from X to $\hat{X}$. Such a Markov kernel leads to a joint distribution on $X\times \hat{X}$ with marginal distribution *P* on X. One may try to minimize (3) for joint distributions and this problem is often easier to solve than finding the optimal quantizer.

The main result in rate distortion theory is that under suitable regularity conditions, we have that the joint distribution on $\left(X,\hat{X}\right)$ that optimizes (3) for n=1 is also optimal for sequences, and the infimum of (3) for joint distributions equals the infimum of (3) for quantizers. In addition, the joint distribution that minimizes (3) is essentially uniquely determined by the slope *s*.

### 1.2. Lossy Source Coding and Statistics

As pointed out in [31], typically, the mathematical formula for distortion does not coincide with our perception of distortion of images. Actually, the mathematical formulas for distortion may be even more relevant for applications in statistics than in image compression.

Before any traditional statistical analysis, data have to be collected. During this data collection phase, data are compressed in the sense that not all observations are stored. Irrelevant aspects of the observations are excluded and data are grouped into bins. If data consist of digital photos, then data are compressed using the data compression algorithms used for digital image compression. With the enormous amount of data generated, modern techniques such as compressed sensing have achieved popularity, because of the increased awareness that some systematic compression should be performed already before the data are stored [32]. Thus, some kind of data compression will always proceed a statistical analysis.

Applications of ideas from rate distortion theory for testing Goodness-of-Fit have been studied in [33,34]. For testing Goodness-of-Fit, we face the problem that data are discrete while the null-hypothesis may claim that the distribution is continuous. Therefore, one has to place data into bins and compare with the probability of these bins under the null-hypothesis. With a distortion function, there is a unique way of making a joint distribution, which can replace the quantization given by bins. After both the empirical distribution and the distribution given by the null-hypothesis have been compressed by the Markov kernel corresponding to the optimal joint distribution, we can use information divergence to compare the compressed distributions.

The purpose of the present paper is to demonstrate that rate distortion theory can also handle a variety of other statistical issues than testing Goodness-of-Fit. For such statistical application, we use the term *rate distortion analysis*. A major advantage of rate distortion analysis is that it is descriptive in nature, which implies that we only need to make minimal assumptions about how the data were generated.

### 1.3. Calculations in the R Program

When we have specified a source alphabet, source distribution, reconstruction alphabet, and distortion function, we need to calculate approximations to the rate distortion function as well as approximations to the optimal joint distributions. These calculations are done with the R program. There was a rate distortion package for the R program [35], but that package has not been maintained. In addition, we need extra features for the type of analysis explained in the present paper. For these reasons, we have developed a new package for R version 4.1.2 for solving rate distortion problems. The software is developed as a general purpose package and incorporates the Blahut–Arimoto algorithm and the package is still in development, but the present version is available online [36]. When fully developed and documented, the package will be uploaded to CRAN. The dataset involving early Islamic mosques has been analyzed using an R worksheet that can be downloaded [37].

In our calculations, the source alphabets are finite. A continuous source alphabet will be approximated by large discrete samples from the continuous distribution. The reconstruction alphabets will be continuous with one or more real-valued parameters. We run the following algorithm in order to calculate the rate distortion function.

1. We create a number of random probability vectors over the source alphabet. These probability vectors are chosen according to a Dirichlet distribution.
2. For each probability vector over the source alphabet, the optimal reconstruction point for these weights is calculated using the Nelder–Mead algorithm, which is a general purpose optimization algorithm built into the R program.
3. With these reconstruction points, we run the Blahut–Arimoto algorithm (see [38] and [30] (Section 13.8)) and obtain a joint distribution on the source alphabet and the reconstruction alphabet.
4. If one of the reconstruction points has probability close to zero, it is removed.
5. If the conditional distributions of source points given two different reconstruction points are close together, then one of the reconstruction points is removed.
6. For each reconstruction point, we replace the reconstruction point by a new reconstruction point that is optimal with respect to the joint distribution, and go back to step 3.

We iterate the steps 3 to 6 until running 3 to 6 gives an improvement of the rate below a certain threshold. The Blahut–Arimoto algorithm in step 3 is iterated until the improvement is below a threshold that is 1/10 of the threshold used as stopping rule for running the loop from 3 to 6.

The reason for choosing a Dirichlet prior in step 1 is that Jeffreys prior is a Dirichlet distribution with parameters 1/2. The reason why Jeffreys prior is of interest is that small information balls with a fixed radius have approximately the same probability with respect to Jeffreys prior. In this sense, Jeffreys prior is like a uniform distribution if the distance between distributions is measured by information divergence. The parameters of the Dirichlet distribution may be fine-tuned in order to make the algorithm run faster.

### 1.4. Organization of the Paper

We illustrate the use of rate distortion analysis by cluster analysis, analyzing Gaussian mixture models, regression for the datasets known as Anscombe’s quartet, and a dataset related to the orientation of early Islamic mosques. These datasets and models are of very different complexity, and the method presented illustrates the generality of our approach and how insight form one model may be migrated to other models.

The rest of the paper is organized as follows. In Section 2, we apply rate distortion analysis for clustering with two examples illustrating determination of the number of clusters and hierarchical clustering.

In Section 3, we demonstrate how the rate distortion analysis works on a Gaussian model, a Gaussian mixture model, and a Gaussian model with an outlier. If the distortion is decreased, the Gaussian mixture will first bifurcate into Gaussian models and when the distortion level gets below the variance of the individual Gaussian distributions, the reconstruction points will experience bifurcations. The same structure will be seen if a dataset contains an outlier except that one of the reconstruction points will be associated only with a single data point.

In Section 4, we analyze linear regression based on four datasets known as Anscombe’s quartet. These are standard datasets used to illustrate that scatter plots can reveal information of the dataset that the most common statistics will not reveal. It is demonstrated that the rate distortion analysis can detect some of the same issues that are usually identified by scatter plots.

In Section 5, we analyze a non-trivial dataset containing measurements of orientations of early Islamic mosques. After providing the historical background, the dataset is analyzed using rate distortion theory. The points on earth, which appear to have determined the direction of prayer in the sense that they give the best fit, have been located. First, some outliers have been identified according to the same criteria as we saw in Section 2, Section 3 and Section 4. Then, the relevant distortion level is determined in the same way as the variance of the components of the Gaussian mixture model was identified in Section 3. Then, the rate distortion analysis is combined with re-sampling in order to obtain confidence regions. Finally, the results are checked using cross-validation. One can use the results to formulate traditional statistical models about the mechanisms that may have generated the data, and one may use traditional estimation and testing techniques for such models, but this is outside the scope of the present article.

The main problem in using the rate distortion analysis for descriptive statistics is the determination of the relevant rate. This issue is discussed briefly in Section 6.

## 2. Cluster Analysis

Identification of clusters in a dataset may be viewed as a special example of rate distortion analysis where the source alphabet equals the reconstruction alphabet. In cluster analysis, the distortion function is often based on a function of a metric, and the use of the squared Euclidean distance is very common [39]. Quantizers in rate distortion theory correspond to hard clustering and joint distributions in rate distortion theory correspond to the use of soft clusters. If all clusters are approximately of the same size, then the rate of a quantizer or a joint distribution corresponds approximately to the logarithm of the number of clusters.

### 2.1. Centroid-Based Clustering

**Figure 1:**
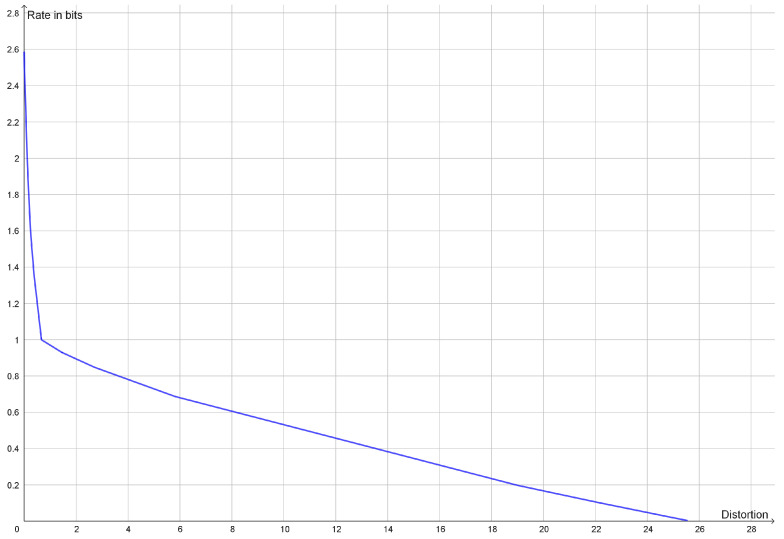
Plot of the rate distortion function of the dataset (−6, −5, −4, 4, 5, 6) with squared Euclidean distance as distortion function. Since the two clusters have the same variance, the elbow is quite prominent at a rate of 1 bit.

This type of clustering has a vector space as source alphabet. The goal is typically to minimize the average of the squared Euclidean distance between elements of a cluster and a reconstruction point, but centroid-based clustering works with any Bregman divergences [40]. The reconstruction point that best represents a cluster is the centroid of the cluster. If one is looking for *k* clusters, one should compress to a rate of approximately ln(k). Instead of looking for a specific number of clusters, one may see how well clustering works with different numbers of clusters. If the dataset has a natural cluster size, the rate distortion function will have a prominent elbow as seen in Figure 1. Such elbows are used as a heuristic method to determine a natural cluster size. Compression to rates above the elbow would lead to overfitting and detection of clusters that are present in the dataset.

In traditional cluster analysis, one would make a scree plot, i.e., a plot of the mean distortion against the number of clusters, but plotting the rate rather than the number of clusters just makes the elbow more prominent. An elbow on the graph of the rate distortion function may be characterized as a point where the optimal rate and the optimal distortion is insensitive to small variations to the slope of the function.

### 2.2. Hierarchical Clustering

**Figure 2:**
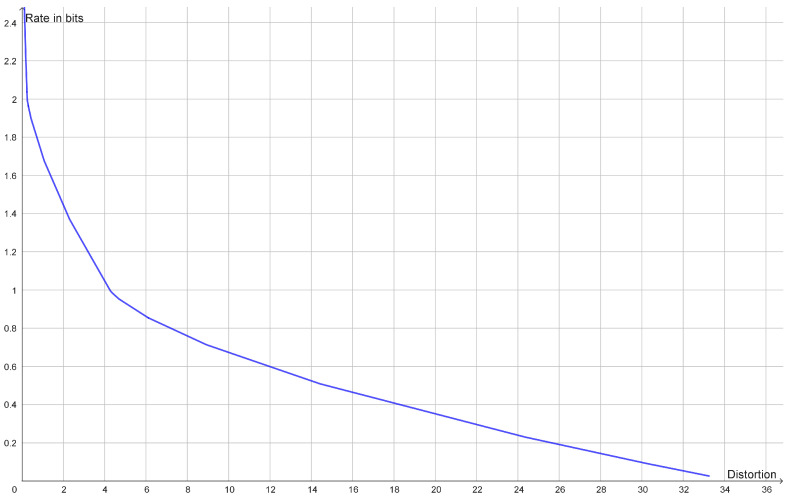
Plot of the rate distortion function of the dataset (−8, −7, −4, −3, 3, 4, 7, 8) with squared Euclidean distance as distortion function. It has an elbow for a rate of 1 bit and another elbow for a rate of 2 bits. This corresponds to two large clusters each consisting of two small clusters.

**Figure 3:**
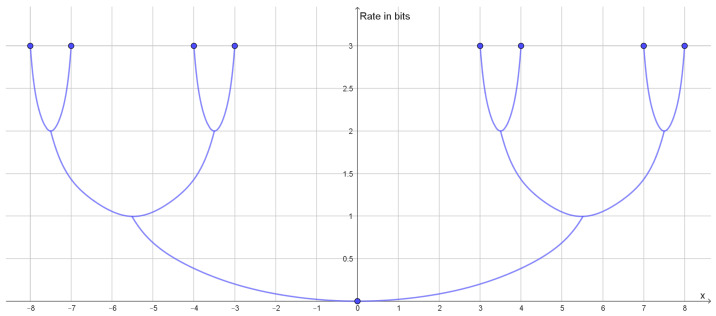
Dendrogram displaying bifurcation structure for reconstruction points for the dataset (−8, −7, −4, −3, 3, 4, 7, 8) with squared Euclidean distance as distortion function. Bifurcations happen for the rate 1 bit and the rate 2 bits where the rate distortion function on Figure 2 has elbows.

If the rate distortion function does not have a prominent elbow, then this is an indication that the data do not contain a single uniform cluster size. This may happen if the clusters have different variance or if the clusters consist of smaller clusters in which case the rate distortion function may have several elbows (Figure 2). In such cases, one may look at how the optimal reconstruction points form a branching tree, which can be identified with a hierarchy of clusters as illustrated in Figure 3. In this example, we see how the elbows of the rate distortion function correspond to the bifurcations of the reconstruction points. In general, the bifurcations of the reconstruction points will be easier to identify than elbows of the rate distortion function.

## 3. Gaussian Mixture Models

A Gaussian mixture model consists of a mixture of Gaussian distributions. For simplicity, we assume that the Gaussian distributions all have the same variance. Modeling by Gaussian mixture models is essentially the same as centroid-based clustering with squared Euclidean distance as distortion function, and it belongs to the type of clustering called *density-based clustering*. Actually, if clustering is based on densities from an exponential family, then the density clustering is equivalent to clustering with a Bregman divergence as distortion function [40,41].

We may think of a Gaussian mixture model as a signal given by a discrete random variable *X* plus some Gaussian noise given by $Z\;\;∼\;\;N\left(0,\sigma^{2}\right)$, where *Z* is independent of X. The output is

(4) Y=X+Z.

**Figure 4:**
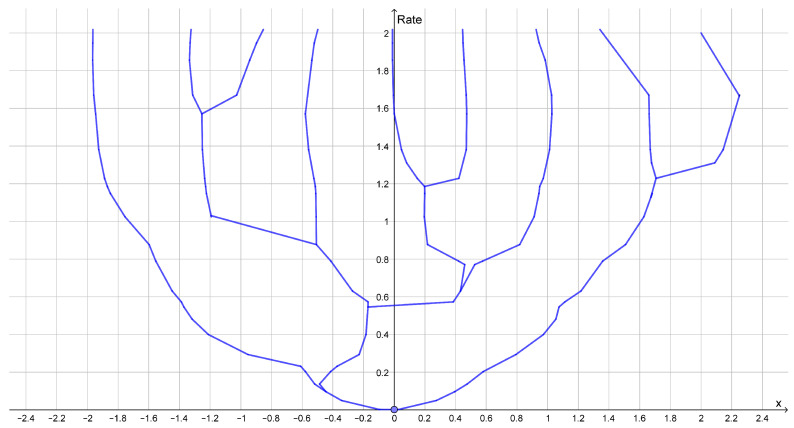
Dendrogram illustrating bifurcation of the reconstruction points for a sample of 100 points samples from a standard Gaussian distribution.

If the standard deviation of the noise is much less than the distance between the different possible values of *X*, then $H\left(X\right)\approx I\left(X,Y\right)$ and the situation becomes very simple to analyze. At a rate below $I\left(X,Y\right)$, compression of *Y* can be done by mapping *Y* into *X* by a maximum likelihood estimate and, then, compression of the estimated value of X. At rates higher than $I\left(X,Y\right)$, compression of *Y* can be done by first mapping *Y* into *X* by a maximum likelihood estimate and, then, compression of Y−X, which is approximately Gaussian. Since Y−X is Gaussian, the optimal compression has a continuous set of reconstruction points, but for real datasets, the slightest deviation from being a Gaussian will lead to a discrete set of reconstruction points as illustrated in Figure 4.

**Figure 5:**
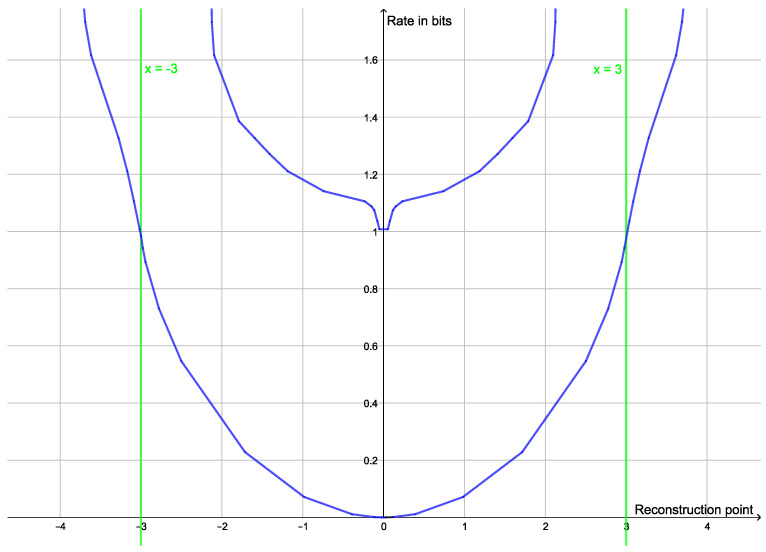
Here, 200 data points are compressed. Of these, 100 were generated by $N\left(-3,1\right)$ and 100 were generated by $N\left(3,1\right).$ This corresponds to a signal of *X* that is a binary variable which is uniformly distributed on $\left\{-3,3\right\}$ and Gaussian noise $Z\;\;∼\;\;N\left(0,1\right).$ The rate of the signal is 1 bit. At rates below 1 bit, the values of Y=X+Z are compressed into two reconstruction points lying symmetrically around 0. At rate 1 bit, we recover the signal X=±3. At higher rates, we start to compress the noise. A reconstruction point at 0 bifurcates into 2 reconstruction points that move towards ±3. The situation stabilizes when the two reconstruction points around 3 (and the two around −3) are at approximately the same distance. At higher rates, more and more new reconstruction points will emerge.

If we do not know σ, we can detect it by mapping the reconstruction points at different rates. Above a certain rate, we start to compress the noise and new reconstruction points will emerge. At this rate, $\sigma^{2}$ will equal the distortion level. This is illustrated in Figure 5.

If σ is not small compared with the difference between the values of *X*, then the transition between compressing the signal and compressing the noise may not be as clear.

**Figure 6:**
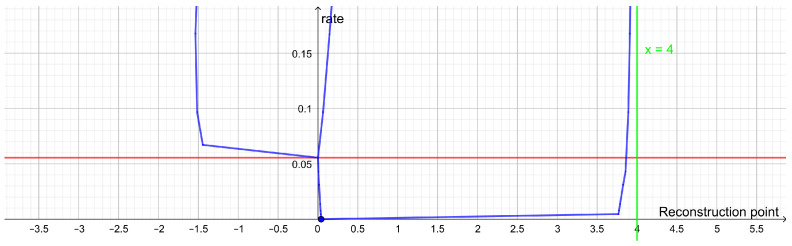
Dendrogram for a dataset containing 100 data points generated by $N\left(0,1\right)$ and an outlier at x=4. The red line indicates a rate of value $\frac{100}{101}\ln\left(\frac{101}{100}\right)+\frac{1}{101}\ln\left(\frac{101}{1}\right)$ corresponding to a distortion of value 1. At higher rate levels, the branches to the left will experience further bifurcations while the branch to the right will stay stable and get closer and closer to the line x=4.

The rate distortion analysis is useful for detection of outliers. This is illustrated in Figure 6. The notion of outliers has been much debated and there are different criteria for declaring outliers and different criteria for how to handle outliers. Criteria for declaring outliers are developed for specific parametric families of probability measures. As we shall see, the rate distortion analysis provides us with a tool that can be used to detect outliers across a broad set of models. In this paper, we only provide examples. A thorough discussion would require a review of the whole literature on outlier detection, and we hope to cover this in a future paper.

As we can see, the rate distortion analysis falls into the category of robust statistics, because the method also works when part of the data do not belong to a single parametric family. A more traditional statistical approach would be to use some kind of non-parametric statistic. In a sense, the rate distortion approach is somewhere between a parametric approach and a non-parametric approach. The parametric aspects are given by the choice of the distortion function and the non-parametric aspects of the method are given by the use of mixture models.

## 4. Anscombe’s Quartet

Anscombe’s quartet consists of four datasets each containing 11 points with two coordinates $\left(x,y\right).$ The four datasets can be seen in Table 1 The datasets all have the same mean value of *x* and y, the same sample variance of *x*, and the same correlation between *x* and *y*, and the same optimal regression line. Scatter plots for the four datasets will reveal that linear regression is only a relevant method for the first dataset. These datasets were created by F. Anscombe [42] to illustrate that graphical illustrations may reveal details that are not captured by the simple descriptive statistics often used for linear regression. We analyze the four datasets using rate distortion analysis and see to what extent the first dataset singles out.

Our source letters will be points $\left(x,y\right)$ from the dataset. The reconstruction letters will be linear models y=ax+b that we identify with points $\left(a,b\right).$ As distortion function, we use the squared Euclidean distance measured in the vertical direction so that

(5) $$d\left(\left(x,y\right),\left(a,b\right)\right)={\left(y-\left(ax+b\right)\right)}^{2}.$$

The distortion function corresponds to the usual regression model

(6) Y=aX+b+Z

where *Z* denotes Gaussian noise independent of *X* with mean zero.

Before we look at the rate distortion analysis of Anscombe’s quartet, we look at the theoretical problem of optimal compression of $\left(X,Y\right)$ when Y=αX+β+Z, where $Z∼N\left(0,\sigma^{2}\right)$ and *Z* is independent of *X*. We have

(7) $$\begin{array}{cc} d\left(\left(X,Y\right),\left(a,b\right)\right) & ={\left(Y-\left(aX+b\right)\right)}^{2}\\ \; & ={\left(\alpha X+\beta +Z-\left(aX+b\right)\right)}^{2}\\ \; & ={\left(Z-\left(\left(a-\alpha \right)X+b-\beta \right)\right)}^{2}. \end{array}$$

If we choose a=α and choose *b* so that b−β is the optimal quantizer of *Z*, then we achieve a rate that lies on the rate distortion curve for compressing Z. The rate distortion function of *Z* is given by

(8) $$R\left(d\right)=\left\{\begin{array}{cc} \frac{1}{2}\ln\left(\frac{\sigma^{2}}{d}\right), & \mathrm{for}0<d\leq \sigma^{2};\\ 0, & \mathrm{for}d>\sigma^{2}; \end{array}\right.$$

where *d* is the distortion and $R\left(d\right)$ is the minimal rate at this distortion [30] (Theorem 13.3.2).

Let $\left(a,b\right)$ be random variables coupled with $\left(X,Y\right).$ Then,

(9) $$E\left[d\left(\left(X,Y\right),\left(a,b\right)\right)\right]=E\left[{\left(Z-\left(\left(a-\alpha \right)X+b-\beta \right)\right)}^{2}\right]$$

and

(10) $$\begin{array}{cc} I\left(\left(X,Y\right),\left(a,b\right)\right) & =I\left(X,\left(a,b\right)\right)+I\left(\left.Y,\left(a,b\right)\right|X\right)\\ \; & \geq I\left(\left.Y,\left(a,b\right)\right|X\right)\\ \; & =I\left(\left.\alpha X+\beta +Z,\left(a,b\right)\right|X\right)\\ \; & =I\left(\left.Z,\left(a,b\right)\right|X\right). \end{array}$$

According to the data processing inequality, we have

(11) $$\begin{array}{cc} I\left(\left(X,Y\right),\left(a,b\right)\right) & \geq I\left(\left.Z,\left(a-\alpha \right)X+b-\beta \right|X\right)\\ \; & =E\left[I\left(\left.Z,\left(a-\alpha \right)x+b-\beta \right|x=X\right)\right]\\ \; & \geq E\left[R\left(E\left[\left.{\left(Z-\left(\left(a-\alpha \right)X+b-\beta \right)\right)}^{2}\right|X\right]\right)\right]. \end{array}$$

The rate distortion function *R* is convex, so we can use Jensen’s inequality to get

(12) $$\begin{array}{cc} I\left(\left(X,Y\right),\left(a,b\right)\right) & \geq R\left(E\left[E\left[\left.{\left(Z-\left(\left(a-\alpha \right)X+b-\beta \right)\right)}^{2}\right|X\right]\right]\right)\\ \; & =R\left(E\left[{\left(Z-\left(\left(a-\alpha \right)X+b-\beta \right)\right)}^{2}\right]\right). \end{array}$$

Hence, the optimal quantization consists of choosing $\hat{a}=\alpha$ and letting $\hat{b}$ be the optimal quantization of the Gaussian random variable Z+β. Thus, the optimal quantization is a mixture of parallel lines $y=\hat{a}x+\hat{b}$ with slope $\hat{a}=\alpha$ and $\hat{b}$ equals to β plus the optimal quantization of the residuals $Z=Y-\left(\alpha x+\beta \right).$

### 4.1. First Dataset

**Figure 7:**
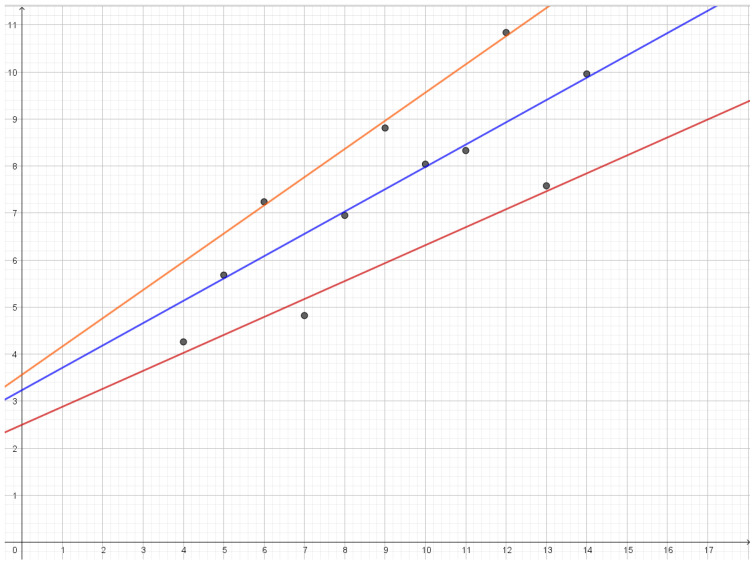
Reconstruction lines for the first dataset in Anscombe’s quartet. As the rate is lowered the orange line and the red line will move towards the blue line.

In the first dataset, we have that the residuals are approximately Gaussian. At a rate of 1.067, we obtain three reconstruction points and an almost perfect fit with the data with a mean distortion of 0.024. As we see in Figure 7, reconstruction points are lines that are almost parallel. As we lower the rate, these lines slowly move closer together and the middle line gets less and less weight until they all merge into a single line with mean distortion 1.12.

### 4.2. Second Dataset

**Figure 8:**
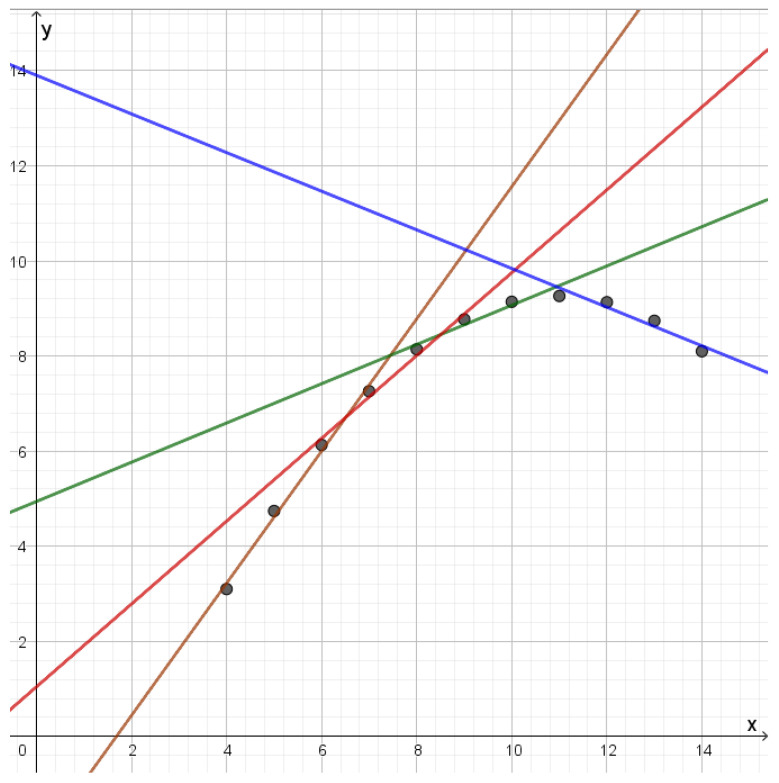
Reconstruction lines for the second dataset in Anscombe’s quartet. The different lines each approximate a certain part of the data set. Since the points lie on a parabola the brown line has a slope that is quite different from the slope of the blue line, while the red line and the green line represent slopes that are in between.

The second dataset consists of points lying on a parabola. The optimal reconstruction points will be lines that each give a good approximation to a part of the parabola as illustrated in Figure 8. These lines will have very different slopes depending on which part of the parabola they approximate. As one compresses, more middle lines will disappear and one is left with two lines with very different slopes. Further compression will gradually merge lines into a single line.

### 4.3. Third Dataset

**Figure 9:**
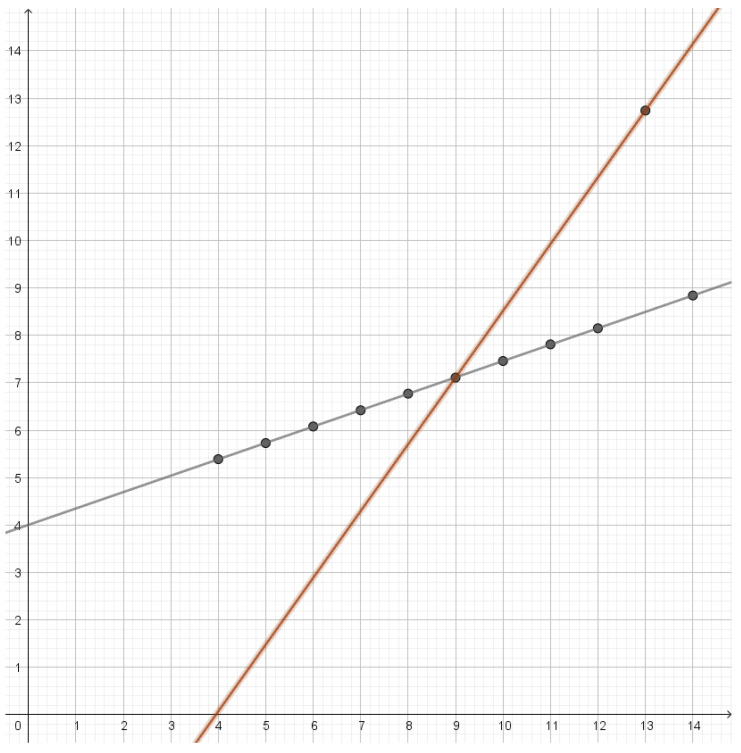
Reconstruction lines for the third dataset in Anscombe’s quartet. All the points except the outlier are on the black line. The brown line goes through the outlier and the center of mass of the rest of the points.

In this dataset, the points lie on a line except for an outlier. There will only be two active reconstruction letters where one corresponds to the straight line through most of the points and the other line goes approximately through the outlier and the center of mass as illustrated in Figure 9. As the rate is decreased below $H\left(\frac{10}{11},\frac{1}{11}\right)$, the line through most of the points will only change slowly. The line through the outlier will first rotate towards the other line and eventually lose weight until the first line has all the weight.

### 4.4. Fourth Dataset

**Figure 10:**
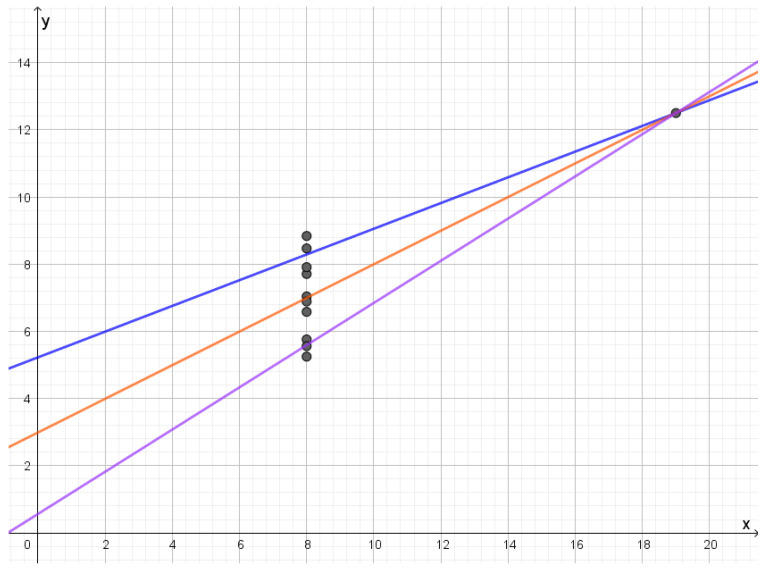
Reconstruction lines for the fourth dataset in Anscombe’s quartet. In this case all the lines go through the outlier. If the rate is lowered the violet line and the blue line will move towards the blue line.

In the fourth dataset, all the points lie on a vertical line except for a separate point. The optimal reconstruction letters will be lines through separate points, as seen in Figure 10. Compression will be compression of the *y*-values of the points on the vertical line. If they follow a Gaussian distribution, the structure of the reconstruction points will be like compressing a Gaussian distribution. In this dataset, the outlier is quite far away from the other points, so the slopes of the lines are not that different. If the outlier was closer to the other points or if the dataset had been larger, one would get reconstruction letters with a larger variety of slopes, which would make the result of the rate distortion analysis much more distinct from the rate distortion analysis of the first dataset.

The four datasets in Anscombe’s quartet have the same optimal regression line corresponding to a compression into a single reconstruction letter. Anscombe’s point was that the different datasets would be easy to tell apart by scatter plots. Here, we have seen that they also behave quite differently with respect to a rate distortion analysis; so, this method could be used as an alternative to the scatter plots. In particular, the rate distortion analysis can be used in higher dimensions where scatter plots are not possible.

## 5. Analysis of Orientation of Early Mosques

A database with information on the orientation of 160 early mosques has been compiled by D. Gibson [43]. We use the data from this database except that we have excluded the ancient mosque in Aqaba where it is unclear which wall was the qibla wall (this mosque has also been excluded from a later version of the database). We will not go into a discussion of the integrity of the data, but at the end of this section, we shortly compare the results with results from a newer version of the database. A statistical analysis of these data has previously been performed and discussed in [44,45].

### 5.1. Historical Background

According to the Islamic traditions, Islam was founded in 622 CE, but the history of early Islam was only written down several hundred years later. According to these traditions, the Muslims were praying facing Jerusalem until Prophet Muhammad in 624 CE received a revelation commanding him to face towards the Sacred Mosque (Mashid al-Ḥarām). Today, Muslims pray facing Mecca where Mashid al-Ḥarām is located. Inside the Sacred Mosque, there is a building called the Ka’ba, which is at the center of the spiritual life of the Muslims. Our oldest reference to Mashid al-Ḥarām is a rock inscription that mentions that it was built (or rebuilt) in Mecca in 698 CE [46] (p. 111).

The Qur’an (Q2: 143–144) states that the direction of prayer (qibla) should be towards Mashid al-Ḥarām, but it is not mentioned where Mashid al-Ḥarām is located. The Ka’ba is mentioned as a destiny of pilgrimage, but it is not mentioned that the Ka’ba is within Mashid al-Ḥarām. Around 700 CE, the Christian author Jacob of Edessa wrote that the Arabs pray towards the Ka’ba, but he does not describe where the Ka’ba was located. Instead, he explains that the Arabs in Egypt face east, and the Arabs in Kufa pray facing west [47]. This does not fit with a Ka’ba in Mecca, but it is known that there were a number of ka’bas at different locations in Arabia [48] (p. 24).

Early Muslim scholars were aware that many of the oldest mosques did not face Mecca. One theory is that the early Muslims did not know the exact direction towards Mecca. Given their ability to navigate through the desert, this seems less likely [49]. There are a lot of indications that Islam has its origin in northwestern Arabia rather than in the area around Mecca [50,51], and the earliest reference to Mecca outside the Qur’an dates as late as 743 CE [52]. There is even an old theory that Petra in Jordan was the birthplace of Prophet Muhammad rather than Mecca [53].

We use orientations of old mosques to provide information on the qiblas used during the formative years of Islam. Typically, a mosque has a long qibla wall with a mihrab (prayer niche) in the middle. Muslims face the mihrab and qibla wall when praying. Many early mosques have a qibla that appears to be inconsistent with a direction facing Mecca.

The Islamic traditions were written during the Abbasid dynasty and, before that dynasty came into power, there had been several civil wars. In the written accounts, the authors openly admit that they have been selective in their choice of narrative. Since the written accounts are late and biased towards the ruling Abbasid dynasty, it is very difficult to judge which parts of these accounts are historically sound. Here, we restrict our attention to the period before the Abbasid revolution in the year 750 CE where the Abbasid dynasty came into power. We subdivide the period in an early period from 622CE to the reforms of the Umayyad Caliph ’Abd al-Malik around 700 CE and a late period from 700 CE to the Abbasid revolution in 750 CE.

### 5.2. Distortion

In this paper, *qibla* is a theory that assigns a certain bearing to each possible location of a mosque. We restrict our attention to potential qiblas that face certain points. Thus, our reconstruction letters are geographical points on earth or on a flat earth approximation of the area. We compare the qibla bearing with the measured orientation of an ancient site. Both the qibla bearing and the orientation can be given as a number of degrees measured clockwise from geographical north. Normally, the orientation will deviate from the qibla bearing which was intended by the people who built the mosque. Four main reasons for this are:

1. The architect may not have been able to determine the qibla bearing exactly.
2. Local obstacles or other practical problems may have influenced the orientation of the site.
3. The original structure may now be a ruin, or it has been rebuilt so that the original orientation is difficult to determine.
4. Sometimes, it is difficult to measure the orientation as discussed in [49].

We have to quantify how much the orientation *o* deviates from the qibla bearing *b* of the site. As distortion function, we use

(13) $$\mathrm{versin}\left(o-b\right)=1-\cos\left(o-b\right).$$

This is the standard method for measuring distortion (also called dispersion) in directional statistics [54] (Section 2.3). The mean of (13) is the *circular variance* Var, which can be translated to *circular standard deviation* σ using

(14) $$\sigma =\frac{360^{\circ }}{2\pi }{\left(-2\ln\left(1-Var\right)\right)}^{1\;/\;2}.$$

The distribution with specified mean direction and specified circular standard deviation that maximizes entropy is a von Mises distribution [55]. Thus, using $\mathrm{versin}\left(o-b\right)$ to calculate distortion corresponds to using the von Mises distributions as our basic error model.

### 5.3. Test of Great Circles vs. Rhumb Lines

D. Gibson has compared the orientations of the mosques with the geodesic directions [49], but he has been criticized that calculations of great circles were not developed in the formative years of Islam [56]. Calculations based on great circles involve trigonometric formulas and astronomical observations that were developed by Muslim scholars later than the period that is the focus of this paper, but, in principle, a bearing may be determined by other means than calculations. Instead of using great circles, they may have used rhumb lines. For our analysis, bearings based on rhumb lines have the advantage that mosques that have the same orientation can be represented by a very distant reconstruction point.

The question is whether we should base the subsequent calculations on bearings calculated along great circles taking the curvature of the earth into account or whether we should calculate bearings along rhumb lines corresponding to a flat earth. Formally, a statistical test is a binary decision based on data; so, we should test great circles versus rhumb lines. Our decision criterion is simple: we choose the model that gives the best compression.

**Figure 11:**
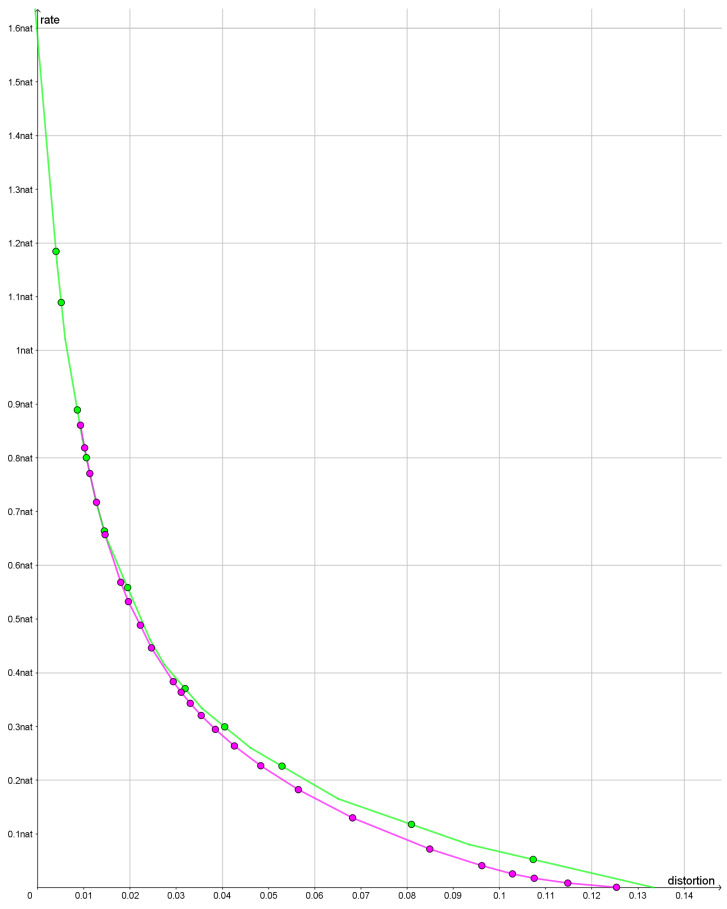
The green polyline is a lower bound on the rate distortion function when we use bearings along great circles. The purple polyline is an upper bound on the rate distortion function when the bearings are calculated along rhumb lines. The dots are points where rate and distortion have been calculated.

In Figure 11, we compare the rate distortion curve when the bearings are based on great circles with the rate distortion curve based on bearings along rhumb lines. For the great circle distortion, we have plotted a lower bound based on the tangents to the rate distortion curve. For the distortion based on the rhumb lines, we have plotted an upper bound by plotting the cords between achievable rate distortion pairs.

For rates less than approximately 0.3 nats using a distortion based on rhumb lines, we find smaller values of the rate distortion function than for the distortion based on great circles. For greater rates, the two curves are very close together. The reason that two distortion functions are so similar for high rates appears to be that the optimal reconstruction points bifurcate and that the bifurcations tend to blur out the difference between the two distortion functions.

Since the bearings based on rhumb lines give a slightly better fit with data, we use bearings based on rhumb lines in the rest of this paper.

### 5.4. Outlier Detection for Sites before 700 CE

First, we make a rate distortion analysis on the set of the 19 sites that are dated prior to the year 700 CE. In order to identify outliers, we should see of if any of the reconstruction points are associated with just one or a few mosques. The rate distortion curve is parameterized by its slope s. For each slope *s*, we obtain a list of reconstruction points and a joint distribution of sites and reconstruction points. Both the reconstruction points and the joint distribution will depend on the rate, but the individual reconstruction points are quite robust to changes in the rate. If we increase the rate, the reconstruction point will split into many reconstruction points, each with little weight. If we decrease the rate, then reconstruction points will merge together. It requires a little experimentation to find a rate that gives an interesting result. Here, we use the slope s=−83.

If we compress with slope s=−83, then we obtain a mean distortion of 0.00395 corresponding to a circular standard deviation of $5.2^{\circ }.$ At this slope, the rate is 0.4840 nats. At this rate, we obtain the three reconstruction points listed in Table 2. Slightly higher rates will cause one of the reconstruction points labeled Pe to bifurcate into reconstruction points very close to the reconstruction point Pe. The other two reconstruction points are associated with very few sites and, for this reason, they will not bifurcate if the rate is increased.

In order to identify these reconstruction points, we look at Table 3 where the conditional probabilities of the reconstruction points are listed for each site.

First, we observe that the Sidi Ghanem mosque is the only mosque that has a significant contribution to the reconstruction point SG. The mosque has been rebuilt many times, and it is not clear which wall was the original qibla wall of this mosque [49]. Compared with the rest of the dataset, we consider Sidi Ghanem as an outlier and we remove it from the dataset.

**Figure 12:**
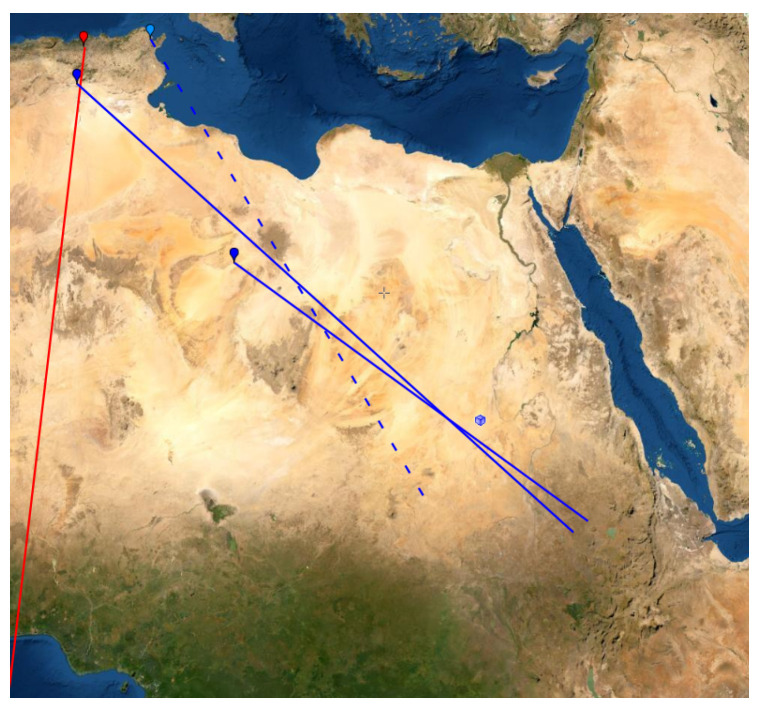
The red marker and line indicate the Sidi Ghanem Mosque and its orientation. The blue markers and blue full lines indicate the locations and bearings of the Sidi ’Ukba Graveyard and the Zawailah mosque. The blue square is the optimal reconstruction point of cluster Ma. The reconstruction point Ma is not at the intersection of the blue lines because there are also some small contributions from sites in the Levant. The blue marker with the dashed line illustrates the only mosque in Magreb between 700 CE and 750 CE.

If the Sidi Ghanem mosque is removed from the dataset, the Graveyard of Sidi ’Ukba will get about 99.9% probability of the reconstruction point Ma and 0.1% probability of the reconstruction point Pe. Thus, the reconstruction point Ma essentially only has contributions from the Graveyard of Sidi ’Ukba and the Zawaila Congressional Mosque. The rest of the sites only gives marginal contributions to the reconstruction point Ma. These two sites and Sidi Ghanem all lie in Magreb, i.e., North Africa to west of Egypt (see Figure 12). Gibson has classified all sites in his database in Magreb as having the “parallel qibla” [49]. Since there are only these three sites in the Magreb from this early period, we can only observe that their qiblas are significantly different from the qiblas from the rest of the sites. Here, we consider these three sites as outliers of the dataset, i.e., they are so untypical that we remove them from the dataset and analyze the rest of the dataset without them.

### 5.5. Determination of Rate and Reconstruction Point

When the outliers have been removed and we use s=−68 as the value of the slope, we obtain a single reconstruction point with coordinates $30.1286^{\circ }$ N $35.4170^{\circ }$ E. The mean distortion for these early sites is 0.00481 corresponding to a circular standard deviation of $5.6^{\circ }.$ If the slope is lowered s≤−69, then the optimal reconstruction point starts to bifurcate into a number of optimal reconstruction points that are located very close to each other as illustrated in Table 4. This is a strong indication that compression with s≤−69 leads to compression of the noise rather than the signal.

We may also conclude that the angular standard deviation is about $5.6^{\circ }$ and this result supports the choice of compression of the dataset with outliers as described in the previous subsection. With the outliers included, we obtained a standard deviation of $5.2^{\circ }$ rather than $5.6^{\circ }$. The reason for this difference is that distortion for the reconstruction points Ma and reconstruction point SG in Table 2 is significantly smaller than the distortion of the reconstruction point Pe. One may say that we obtain overfitting if we do not exclude outliers. For instance, the reconstruction point SG is primarily associated with the Sidi Ghanem Mosque, and the reconstruction point is exactly on the line corresponding to the orientation of the mosque. This leaves an extra degree of freedom that is used to adapt to the orientations of some of the other sites even though these other sites may have nothing to do with the orientation of the Sidi Ghanem Mosque.

### 5.6. Calculation of a Descriptive Confidence Region

The use of confidence regions is widely used in statistics, but due to the complexity of the data and the model, we do not have formulas for calculating such confidence regions. Instead, we use bootstrap techniques as described in [57] (Section 5.2) to calculate a region that resembles the well-known notion of a confidence region. The interpretation is closely related to the notion of cross-validation.

The optimal reconstruction point Pe is obtained by minimizing the mean distortion where each of the 16 sites has weight 1. One may argue that a large congressional mosque should have larger weight than a small rural mosque. If a mosque has two qiblas, one may ask if each of the two qiblas should have the same weight as a mosque with a single qibla. One may also ask if a mosque rebuilt with the same qibla should count as one or two. Finally, some may question the dating of some mosques. They may argue that some mosques should be removed from the dataset. One of the main purposes in natural sciences for making controlled experiments is to obtain exchange-ability of the individual results. Exchange-ability implies that all data points should have the same weight. In humanities, we often face the problem that data are not collected by controlled experiments. Therefore, there is no default reason why all sites should have the same weight. We examine what happens if we randomly assign weights to the sites before we calculate the optimal reconstruction point.

The random weights are assigned by re-sampling. From the 16 sites, we sample 16 sites with replacement. In such a bootstrap sample, only about 67% of the original sites will appear, and some sites will appear several times such that the sites in the bootstrap sample will have multiplicities that sum up to 16. This corresponds to assigning random integer weights to the sites. For this bootstrap sample, we find the optimal reconstruction point where the mean distortion is calculated with weights of the sites given by the multiplicity specified by the bootstrap sample. In principle, we should go through all possible re-samples, but instead, we have used a large random sample. Then, we approximate the distribution of reconstruction points by a 2-dimensional Gaussian distribution. Finally, we calculate the ellipse that contains 95% of probability mass of the 2-dimensional Gaussian distribution and this will be our descriptive confidence region.

If a similar procedure is used for a binomial distribution, one will get the formula for calculation of the well-known *z*-interval of the success probability.

**Figure 13:**
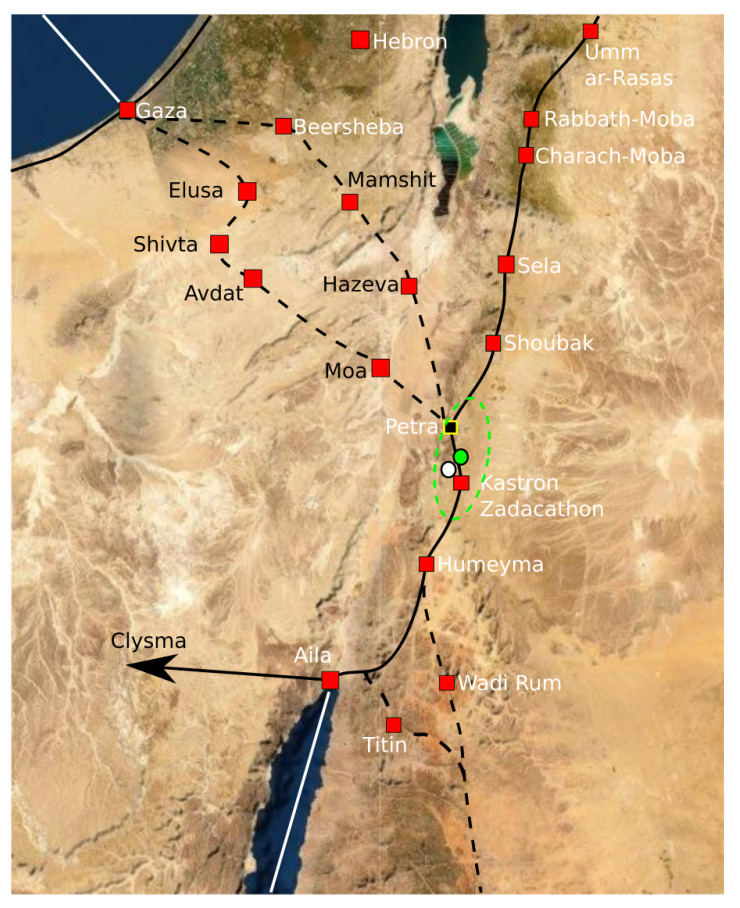
The white dot is the optimal reconstruction point for cluster Pe based on 18 sites. The confidence region is outlined by the green ellipse and its center is marked by a green dot.

Instead of going through all $16^{16}$ ways of re-sampling, we just randomly take 10,000 re-samples and base our calculations on that. Bootstrap re-sampling is implemented by the bootstrap package in the R program. The resulting descriptive confidence region is depicted in Figure 13. The descriptive confidence region is confined around the ancient city of Petra. Inside the region, there are no obvious alternative candidates for an early Islamic qibla.

Around 700 CE, Jacob of Edessa stated that the mhaggriiye (Arabs) pray towards the Ka’ba. Since the optimal reconstruction point is consistent with the text of Jacob of Edessa, we believe that he refers to the qibla associated with cluster Pe. Hence, there must have existed a Ka’ba in the confidence region around the reconstruction point Pe before 700 CE.

As our descriptive confidence method shows, the conclusion will not alter if we remove a few of the sites from the dataset or if we give the sites slightly different weights. One problem about this analysis is that it may suffer from selection bias. In this period, the mosques do not have mihrabs; so, the identification of whether a site has a qibla may have been effected by how the building is oriented compared with Petra and Mecca. For this reason, we use the later mosques to cross-validate our temporary conclusion that early Muslims outside Magreb used Petra as qibla.

### 5.7. Cross-Validation

The analysis of mosques from before 700 CE suggests that the early Muslims were able to determine the qibla with a mean distortion of 0.00481 corresponding to a circular standard deviation of $5.6^{\circ }$ Now, we can compress the data involving all sites before 750 CE using the same distortion level. A short summary of the results is as follows:

- All the mosques in the Magreb appear as outliers and are removed from the dataset.
- The reconstruction point Pe associated with Petra appears again with approximately the same coordinates and the same confidence region.
- A reconstruction point Je appears with Jerusalem as qibla. Only a single mosque at Qasr Tuba is associated with this qibla with high confidence. A few other mosques could also have had this qibla.
- Two new reconstruction points emerge south and southeast of Petra. If the slope is increased to s≥−29, then these two reconstruction points merge into a single reconstruction point that we label Ru.

One may conjecture that the early Muslims were, for some unknown reason, not able to determine the bearing to the reconstruction point Ru with the same precision as the mosques facing Petra. The result can be seen in Table 5. With this compression, the distortion of the reconstruction point Ru is 0.01329 corresponding to a circular standard deviation of $9.4^{\circ }.$ The mosques associated with the reconstruction point Ru are approximately the same as the ones that D. Gibson classified as having “the between qibla” [49]. According to Dan Gibson’s theories “the between qibla” was used because of political tensions between the ruling Umayyad dynasty and the Abbas family that would later establish the Abbasid dynasty. According to his theories, the mosques that used “the between qibla” used the bisector or median between the bearing to Petra and the bearing to Mecca.

**Figure 14:**
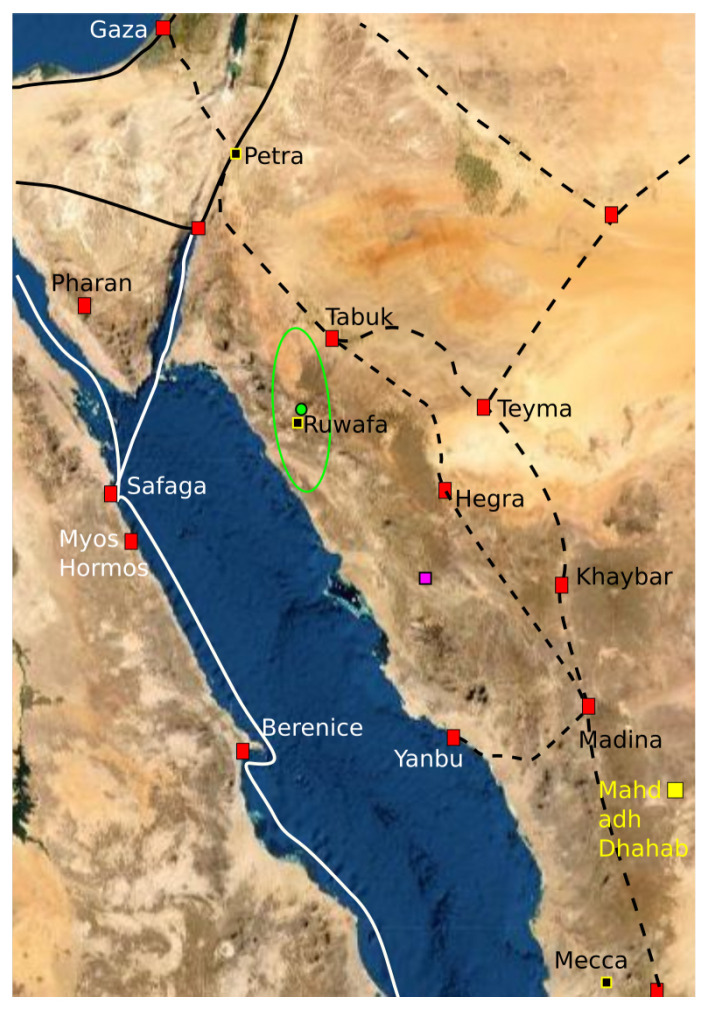
The green curve is placed around the 95% confidence region based on a compression with s=−29. The green dot is the optimal reconstruction point of the cluster Ru. The black square with yellow border is the actual location of the Ruwāfa Temple. The purple marker indicates the midpoint between Petra and Mecca along a great circle.

In order to calculate a confidence region for the reconstruction point Ru, we fix the reconstruction points for the cluster Pe to Petra and fix the reconstruction point of the cluster Je to the Dome of the Rock in Jerusalem. With these reconstruction points fixed, we calculate the optimal reconstruction point of cluster Ru and calculate a confidence region around this point. The confidence region for cluster Ru is illustrated in Figure 14. The confidence region does not contain any ancient settlements, and it does not intersect any of the ancient trade routes. The confidence region does not contain the midpoint between Petra and Mecca as suggested by D. Gibson. The optimal reconstruction point is located at $27.6664^{\circ }$ E 36.2188${}^{\circ }$ N. The only site of archaeological relevance within the confidence region is the Ruwāfa Temple that lies completely isolated in the desert [58] (pp. 44–49) and it is located just a few kilometers from the optimal reconstruction point. The temple has not been excavated, but some interesting inscriptions reveal that it was built around 160 CE for Ilāh (older version of Allāh). The observation that the Ruwāfa Temple is almost at the optimal reconstruction point is new knowledge, and it is still far from clear how this result should be interpreted.

We can test Gibson’s “between qibla” versus our “Ruwāfa qibla” in the same way as we tested great circles versus rhumb lines and the result is that “Ruwāfa qibla” gives a much better description of the data than the “between qibla”.

In January 2022, an updated version of the qibla database was published [59]. This new database can be used to cross-validate our results. The new database includes 204 sites, which means that there are 44 more sites than in the previous database. The new database also contains some corrections to data in the previous database. The analysis of sites prior to 700 CE leads to exactly the same result as before: the earliest mosques face a point very close to Petra except for some outliers in the Magreb. The analysis of sites prior to the Abbasid revolution in the new database has some extra outliers. In addition to the previously identified reconstruction point, there are two new reconstruction points. One of the new reconstruction points is close to Mecca. In the previous database, there were so few sites with this qibla that Mecca could not be singled out as a separate reconstruction point, but this has changed with the increased size of the database.

There is also a new reconstruction point near the city of Aqaba. The sites associated with this reconstruction point are mainly new sites from the Negev Highland in present-day Israel. The emergence of this new reconstruction point and a new reconstruction point near Mecca demonstrates that there has been a selection bias in the 2021 version of the database.

## 6. Discussion

In this paper, we have seen various applications of rate distortion analysis in statistics. The main problem with our application of rate distortion analysis is that we have to choose a distortion level. Different choices of distortion level may in principle lead to different conclusions. For the datasets that we have analyzed in this paper, we found an appropriate distortion level by experimentation with different levels. The distortion is directly related to the specific application. Although this method may work well in many applications, we would like to develop quantitative criteria for the choice of the distortion level.

Since the relevant distortion level will depend on the specific distortion function and the specific application, it may be better to select a distortion level that leads to a certain rate and let this rate be dependent on the sample size. In certain areas of application, we actually have some rules of thumb that may be translated into relations between rate *R* and the sample size n. In testing Goodness-of-Fit, one may divide data into *k* bins and the number of bins should not grow faster than $\frac{n}{\ln\left(n\right)}$ in order to ensure Bahadur efficiency. If we also want the statistics to be asymptotically normal, the number of bins should not grow faster than $n^{1\;/\;2}.$ If we follow the square-root choice where data are distributed into $k=n^{1\;/\;2}$ bins of equal probability, then the rate will be $R=\ln\left(k\right)=\frac{1}{2}\ln\left(n\right).$ Alternatively, the Rice rule suggests $k=2n^{1\;/\;3}$, which translates into $R=\ln2+\frac{1}{3}\ln\left(n\right).$ There are a number of other rules of thumb that each may be translated into the rate distortion framework. We hope that the rate distortion analysis will be instrumental in developing rules of thumb that are less dependent on the specific problem at hand.

## Figures and Tables

**Table 1:** The four datasets that constitute Anscombe’s quartet.

I		II		III		IV	
*x*	*y*	*x*	*y*	*x*	*y*	*x*	*y*
10.0	8.04	10.0	9.14	10.0	7.46	8.0	6.58
8.0	6.95	8.0	8.14	8.0	6.77	8.0	5.76
13.0	7.58	13.0	8.74	13.0	12.74	8.0	7.71
9.0	8.81	9.0	8.77	9.0	7.11	8.0	8.84
11.0	8.33	11.0	9.26	11.0	7.81	8.0	8.47
14.0	9.96	14.0	8.10	14.0	8.84	8.0	7.04
6.0	7.24	6.0	6.13	6.0	6.08	8.0	5.25
4.0	4.26	4.0	3.10	4.0	5.39	19.0	12.50
12.0	10.84	12.0	9.13	12.0	8.15	8.0	5.56
7.0	4.82	7.0	7.26	7.0	6.42	8.0	7.91
5.0	5.68	5.0	4.74	5.0	5.73	8.0	6.89

**Table 2:** Reconstruction points for s=−83. The labels of the reconstruction points are based on the interpretations of the clusters given later in this paper.

Rec. Point	Latitude	Longitude	Weight	Distortion
Pe	$-30.1439^{\circ }$	$-35.4267^{\circ }$	77.16%	0.00446
*Ma*	$-18.5177^{\circ }$	$-28.7456^{\circ }$	15.34%	0.00266
*SG*	$-5.4385^{\circ }$	$-33.4956^{\circ }$	7.50%	0.00134

**Table 3:** Soft classification of sites older than 700 CE. Probabilities are given in percent.

Site	Pe	Ma	SG
Massawa Mosque	100.0	0.0	0.0
Huaisheng Mosque	86.9	0.1	13.0
Hama Great Mosque	91.1	8.9	0.0
Palmyra Congregational	92.2	7.8	0.0
Amr ibn -Al-As	100.0	0.0	0.0
Sidi Ghanem	0.0	0.0	**100.0**
Graveyard of Sidi ’Ukba	0.1	**58.2**	41.7
Qasr Humeima	100.0	0.0	0.0
Zawailah	0.0	**100.0**	0.0
Dome of the Chain	100.0	0.0	0.0
Ka’ba	100.0	0.0	0.0
Qasr El-Bai’j	79.2	29.6	0.0
Um Jimal Later Castellum	84.7	15.3	0.0
Kathisma Church	100.0	0.0	0.0
Qasr Mushash	86.1	13.9	0.0
Seven Sleepers Mosque	92.1	7.9	0.0
Husn Umayyad Mosque	97.4	2.6	0.0
Zeila Qiblatain Mosque (Rt)	100.0	0.0	0.0
Zeila Qiblatain Mosque (Lft)	100.0	0.0	0.0

**Table 4:** Reconstruction points at slope s=−69 for sites dated before 700 CE. Outliers were removed.

Rec. Point	Latitude	Longitude	Weight	Distortion
Pe1	$30.1286^{\circ }$	$35.4170^{\circ }$	99.87%	0.00481
Pe2	$30.5953^{\circ }$	$35.3761^{\circ }$	0.13%	0.00498

**Table 5:** Clusters at slope s=−29 with outliers removed and Petra and Jerusalem fixed.

Rec. Point	Latitude	Longitude	Weight	Distortion
Je	$31.7781^{\circ }$	$35.2353^{\circ }$	2.57%	0.00662
Pe	$30.3289^{\circ }$	$35.4433^{\circ }$	34.01%	0.01134
Ru	$27.6664^{\circ }$	$36.2188^{\circ }$	63.42%	0.01329

## Data Availability

The database with orientations of early mosques is available at https://figshare.com/articles/dataset/Early_Islamic_Qibla_Database_2022/19087784/2 (accessed on 25 December 2022). The database exists in a version from 2021 and most of the calculations in this paper are based on the 2021 version. The 2022 version of the database is used for cross-validation.
